# The women who wrote in the Manicomio Nacional de Leganés after the Spanish Civil War, 1939-1952

**DOI:** 10.1590/S0104-59702024000100022

**Published:** 2024-05-17

**Authors:** Ana Conseglieri, Olga Villasante

**Affiliations:** i Psiquiatra, Hospital Universitario Infanta Cristina. Parla – Madrid – España anaconseglieri@gmail.com; ii Psiquiatra, Hospital Universitario Severo Ochoa. Leganés – Madrid – España olga.villasante@salud.madrid.org

**Keywords:** Psychiatric care, Spanish Civil War aftermath, Correspondence, Women mentally ill, Written culture, Asistencia psiquiátrica, Posguerra española, Correspondencia, Enfermas mentales, Cultura escrita

## Abstract

This article uses the medical records of six women admitted to the Manicomio Nacional de Leganés, Madrid (Spain), in which, in addition to medical notes, there are letters and other personal documents. These unsent letters allow us to read about their complaints towards the institution, as well as to recover the voices of the inmates and their resistance to being treated like insane people. This analysis leads us to explore the double marginalization: being “women” and being “mentally ill”; it also brings us closer to building a story from the patient's point of view. The time frame is Franco’s dictatorship, during which the implementation of a national-Catholic system undoubtedly reinforced the female hegemonic model of the regime.

## Resumen

El presente trabajo utiliza los historiales clínicos de seis mujeres internadas en el Manicomio Nacional de Leganés, Madrid (España), en los que, además de las anotaciones médicas, existen cartas y otros documentos personales. El control sobre estas cartas no enviadas nos permite escuchar las denuncias sobre la institución, así como recuperar las voces de las internas y sus resistencias a ser tratadas como locas. Este análisis nos lleva a explorar la doble marginación: ser “mujeres” y “enfermas mentales” y nos aproxima a construir una historia desde el punto de vista del paciente. El marco temporal es el franquismo, régimen dictatorial que implantó un modelo hegemónico femenino dictado por una moral nacional-católica.

En las últimas décadas se han multiplicado los trabajos que destacan la importancia de los escritos de pacientes como fuentes privilegiadas para el estudio de una historia de la medicina desde "el punto de vista del paciente" (Porter, 1987). La influencia del artículo pionero "The patient's view: doing medical history from below", de Roy Porter (1985), es cada vez mayor en diferentes ámbitos geográficos (Davis, 2001; Condrau, 2007). Desde diversas perspectivas la historiografía ha abordado la escritura en establecimientos cerrados como cárceles o reformatorios (Castillo, Sierra, 2005; Sierra, 2016), así como los textos de personas internadas en instituciones psiquiátricas (Barfoot, Beveridge, 1990; Reaume, 2000). De hecho, las publicaciones de trabajos en esta última línea han proliferado en los últimos años, y aunque se escapa a la extensión de este artículo el poder citarlos todos, señalaremos el libro de Cristina Rivera Garza (2022) *La Castañeda: narrativas dolientes desde el manicomio general, México, 1910-1930*, donde se recogen algunos testimonios de pacientes entresacados de las entrevistas con los médicos del manicomio mexicano. Por otra parte, en relación a las instituciones brasileñas, nombraremos a Yonissa M. Wadi (2010) quien ha analizado la escritura de mujeres como Pierina, una infanticida internada en el Hospício São Pedro, de Porto Alegre, o el relato de la escritora Maura Lopes Cançado. Esta última, hospitalizada entre 1950-1970, plasmó en *Hospício é Deus: diário* su experiencia durante un tercer ingreso en 1959, en el Centro Psiquiátrico Nacional, no Rio de Janeiro, criticando algunas prácticas institucionales (Wadi, 2017).

En España, en el marco del Programa Leganés,[1] se han rescatado las historias clínicas como fuentes de archivo para abordar la historia institucional y acercarnos a una práctica clínica que no siempre se ajustó a "los conocimientos o los paradigmas imperantes" (Huertas, 2001, p.8). En las últimas décadas, el Manicomio Nacional de Santa Isabel de Leganés en Madrid (Peset, 1995; Villasante, 2003) ha sido el eje de numerosas investigaciones que, además de utilizar estos documentos para el análisis de la población manicomial o de las prácticas psiquiátricas (Conseglieri, 2008; Vázquez de la Torre, Villasante, 2016; Candela, Villasante, 2018), han recuperado las cartas y textos de pacientes conservados dentro de los historiales clínicos. Estos escritos, recopilados en *Cartas desde el manicomio: experiencias de internamiento en la Casa de Santa Isabel de Leganés* (Villasante et al., 2018), describen tanto el funcionamiento de la institución o la vida cotidiana en su interior como las experiencias o emociones de sus autoras o autores, ofreciendo diferentes elementos enriquecedores para la historia de la psiquiatría institucional desde la perspectiva de las pacientes (Villasante et al., 2016). A pesar de la importancia de esta documentación personal, tan interesante desde el campo de la subjetividad, debemos señalar la escasez de este tipo de material (cartas, autobiografías, poemas o dibujos) hallado en tan solo 26 de las 615 historias clínicas abiertas entre 1939 y 1952 (Conseglieri, 2013a), de las cuales únicamente seis de ellas corresponden a mujeres.

Estas seis mujeres que ingresaron entre 1940 y 1947 en el citado manicomio son las únicas de quienes conservamos escritura propia y, por ello, nos proponemos recuperarla. Si bien sus biografías no son conocidas como las de Sylvia Plath (2012), Leonora Carrington (2017) o activistas como Kate Millet (2019; Huertas, 2020a), quienes reprodujeron su propio sufrimiento psíquico, consideramos importante rescatar parte de estas vidas anónimas, cuya voz fue silenciada hace más de siete décadas. El presente trabajo trata de analizar no solo el discurso médico plasmado en los documentos clínicos, sino dibujar algunos aspectos del sufrimiento de estas mujeres a través de las cartas, no enviadas, que escribieron a familiares, médicos, o jueces. Así pues, estas misivas que se conservan en los archivos clínicos, van a ser utilizadas como documentos auxiliares en la investigación (Doll Castillo, 2002) y trasladadas, como señala Roxana Pagés-Rangel (1997), al dominio público.

A pesar que el número de casos y la correspondencia son escasas, este análisis se enmarca en una línea historiográfica y epistemológica más amplia centrada en la historia desde el punto de vista de las pacientes que han sido desprovistas de la palabra (Huertas, 2013). La condición de ser mujer, ingresada en una institución psiquiátrica, la enmarca en una doble subalternidad: ser “mujer” y estar “loca” (Huertas, 2020b, p.89-117) ante la cual la disciplina psiquiátrica ha ejercido su propio poder (Chesler, 2019), tal como han analizado García-Díaz y Jiménez-Lucena (2010, 2023) para el caso del Manicomio Provincial de Málaga.

Estas mujeres fueron internadas durante la primera etapa del franquismo (1939-1952), un régimen dictatorial instaurado tras la Guerra Civil Española (1936-1939) que trató de eclipsar los avances realizados durante la Segunda República (Novella, Campos, 2017) imponiendo un ideario femenino que identificaba a la mujer con la maternidad y la domesticidad. Reconocemos las limitaciones de la investigación y no pretendemos adoptar un análisis desde el feminismo; tampoco podemos asegurar que la recuperación de estas voces silenciadas durante décadas no esté condicionada por nuestro lugar como investigadoras, tal como ha reflexionado Spivak (2010). Aún considerando este sesgo, el estudio de los casos de María, Elena, Josefa, Adela, Matilde y Elvira – nombres ficticios – puede contribuir a un conocimiento más amplio de la historia del Manicomio Nacional de Leganés.

Nuestra intención a lo largo de este artículo será, en primer lugar, trazar los seis retazos biográficos de estas mujeres institucionalizadas, asumiendo que son solo descripciones de fragmentos de sus vidas, debido a la existencia de unas historias clínicas psiquiátricas pobres e incompletas. Además, rescataremos parte de sus escritos con el fin de buscar elementos comunes y diferenciales entre ellas, tratando de disminuir la laguna historiográfica en torno a la historia de este grupo subalterno (Tenti, 2012). Por último, nos acercaremos a sus demandas, angustias, preocupaciones y a las denuncias sobre la institución, así como a las resistencias a permanecer en ésta (Huertas, Villasante, 2019), ya que ayudan a conocer aspectos de la asistencia psiquiátrica en España, contribuyendo, de un modo más amplio, al estudio de la locura.

## María, toda una vida en el manicomio

María, nacida en Cáceres, fue, según el psiquiatra, una niña “triste y cobarde, retraída y poco activa”, con una vida marcada por la enfermedad mental de su madre y un tío materno (Historia clínica n.XX-1220 recogida en Villasante et al., 2018, p.165-166).[2] Había sido ingresada por primera vez en 1917, a los 20 años, mostrándose nerviosa a temporadas: unas veces se deprimía y otras parecía “agitada y agresiva, sucia, cantarina y con apetito voraz” (p.166). En 1940, a la edad de 43 años, llegó al Manicomio de Leganés, a instancias de su hermano, como pensionista de primera clase, donde fue diagnosticada de psicosis maniaco-depresiva y tratada con electrochoque, un tratamiento introducido en dicha institución a partir de 1944 (Conseglieri, Villasante, 2021).

Anteriormente había sido ingresada al menos siete veces en un sanatorio regentado por las Hermanas Hospitalarias de San Juan de Dios en Ciempozuelos, Madrid. En su historia clínica, aparecen unas líneas dirigidas a su tío paterno en un formato no habitual de carta, ya que se halla escrito en el evolutivo del propio historial:

> Estoy hablando con el Señor Director y me da permiso para ir a ver a usted y comer muchas cerezas de las buenas y frutas y buen pan. Las primas estarán bien, recibí carta de V., no la contesté por estar delicada [*sic*], me marcho por Oropesa, donde están los primos pasaré por Candeleda de Ávila, y os abrazaré a todos. Ya te contaré todas las pesetas que llevaré a la Iglesia de P.C. de María Madre de Dios, ya te decía que te contaría lo que haría con tu dinero para ir todos a misa del purísimo (Villasante et al., 2018, p.166).

Dada la fecha – 6 de junio de 1946 –, es posible que la carta fuese propiciada por Manuel Peraita, ya que corresponde a la grafía de este médico, director del manicomio entre 1944 y 1949 (Conseglieri, 2013b). La escritura pudo utilizarse a modo de prueba exploratoria para mejorar la comprensión clínica de la paciente, tal como habían promovido, con fines diagnósticos y terapéuticos alienistas franceses como Brierre de Boismont, Luis Marcé (Artières, 1998) o José Salas y Vaca, director de Leganés en las primeras décadas del siglo XX (Candela, Villasante, 2013; Villasante, Candela, 2014).

Las escasas líneas de María aludían al “buen pan”, en contraposición a aquel pan negro típico de la posguerra española con escasa cantidad de trigo. No obstante, en su misiva, se refería a la contribución económica al mantenimiento de la institución religiosa y añoraba estar junto a su familia. Un deseo que mantendría probablemente toda su vida, al permanecer más de 20 años en Leganés sin que ningún familiar se hiciese cargo del cuidado de esta mujer soltera, después trasladada al Manicomio de Zaragoza en 1961.

## Elena: una mujer culta, independiente y atea

La familia de Elena la recordaba como una niña de “carácter alegre, rebelde, aficionada a salir con amigas, lista y capaz de aprender todo fácilmente” (Historia clínica n.XX-1303 recogida en Villasante et al., 2018, p.167-169). De origen madrileña, estudió en un colegio alemán, y aprendió a tocar el piano. Sin embargo, cuando llegó a la pubertad, parece que no se ajustaba al modelo esperado, y su propia familia la describía como “perezosa” e “indiferente hacia las labores propias de su sexo” (Villasante et al., 2018, p.167).[3]

A los 20 años, comenzó a tener conflictos con sus padres, debido a las restricciones que le imponían en sus relaciones con los hombres. A los 26, ingresó en un sanatorio privado (Doctor León) recibiendo piretoterapia con sulfosin,[4] un derivado del azufre utilizado para provocar artificialmente un cuadro febril que supuestamente mejoraba el estado clínico. Además estuvo, entre otros, en el prestigioso sanatorio neuropsiquiátrico del doctor Lafora recibiendo tratamiento malarioterápico (Villasante, 2020),[5] y donde el propio director del centro solicitó su traslado al ya citado sanatorio de Ciempozuelos por conductas inapropiadas hacia los médicos. En este último establecimiento fue tratada por José Miguel

Sacristán[6] y José Salas Martínez (Villasante, 2016),[7] aunque ante el avance del frente, fue trasladada al Sanatorio de San Luis (Palencia) para regresar nuevamente a Ciempozuelos (Madrid) tras la guerra. Su peregrinación por instituciones psiquiátricas no finalizó en Ciempozuelos y aún fue recluida en otro centro privado (Sanatorio del Doctor Esquerdo), y tratada con el coma insulínico.[8] Los médicos describían que los "accesos con rebeldía y obsesiones que giraban, principalmente, en torno a los enamoramientos" con los hombres eran el motivo de ingreso en todos los internamientos (Villasante et al., 2018, p.167).

La primera vez que entró en el Manicomio de Leganés, con 37 años, fue diagnosticada de esquizofrenia mostrándose disconforme, al considerar que habían sido sus padres quienes, como en el resto de ocasiones, le habían recluido por su conducta. El 17 de junio de 1944, Elena acudía al despacho de Peraita, hablando en alemán, y con un rito de subordinación en pos de reparar sus conductas "inapropiadas" como la de "dirigirse a los hombres o llamarles por teléfono", y así regresar a su domicilio (Historia clínica n.XX-1303, dic. 1941, s.p.). En septiembre salió de permiso temporal y posteriormente fue dada de alta, aunque en 1949, regresaba a Leganés una vez más a instancias de su familia.

Las descripciones clínicas del médico incluían características como "plañidera y pueril, … obsesionada por tener novios, llevar una vida independiente buscando un hombre para casarse y querer brillar en sociedad … mismas características en su conducta que han motivado sus distintas reclusiones" (Historia clínica n.XX-1303, dic. 1941, s.p.), todas ellas cualidades alejadas de valores como el sacrificio, la sencillez y la austeridad que el primer franquismo promovía para la mujer (Blasco Herránz, 1997, p.93). Además, sobresalía el componente sexual en el diagnóstico, porque tuvo "contactos sexuales" con sus parejas, lo que ponía "de relieve que su conducta no es normal ni correcta en una señorita" (Historia clínica n.XX-1303, dic. 1941, s.p.). Son comportamientos no convencionales que manifiestan como la locura ha servido como justificación para patologizar diferentes conductas discordantes en las mujeres (Saez, 1979; Showalter, 1985; Basaglia, 1987; Hirshbein, 2010; Miranda, 2019); una cuestión también observada en aquellas cartas escritas desde el antiguo Ospedale Roncati de Bolonia (Montanari, 2015) o en los casos de mujeres "incomodas e insumisas" del periodo victoriano, objeto de reclusión en manicomios, narrados por Sarah Wise (2012). Rivera Garza (2001, p.680) también muestra casos de hijas que atentaban contra las reglas tradicionales en sus relaciones sexuales con los hombres.

La religiosidad de las pacientes plasmada por los médicos en sus historias se muestra como un aspecto importante, como observamos en la siguiente anotación: "A pesar de su previa inclinación por las prácticas religiosas, comenzó a manifestar una marcada aversión atribuida al maltrato de las monjas. En ocasiones, su irreligiosidad llegó a explícitas manifestaciones de ateísmo, negando la existencia de Dios" (Villasante et al., 2018, p.168). No hay que olvidar que el nacionalcatolicismo durante la dictadura fue un pilar fundamental para mantener a la población bajo control y, especialmente, a la mujer (Moreno Seco, 2008, p.275).

De los dos únicos escritos de Elena hallados, uno iba dirigido al médico excusándose por su actitud y pidiendo compasión en un acto desesperado por conseguir su salida.[9]

> Leganés 21 de abril de 1967. Un buen amigo Valentín. Usted debió juzgarme muy mal el último día que usted me habló porque dije dos cosas que no debía haber dicho. …

> No puedo escribirle más, lo único que le pido es que no piense usted mal de mí, pues yo nunca he sido una mujer interesada. Esperando a ver si viene usted pronto para volverle a ver se despide hasta verle otra vez, la casi más desgraciada de sus enfermas. Elena. Si me quiere buscar una colocación yo trabajaré para usted sin cobrar ni un céntimo (Villasante et al., 2018, p.169).

No obstante, y pese a las numerosas altas previas, de este último internamiento ya no salió, permaneciendo el resto de su vida hasta fallecer, a comienzos de 1970.

## Josefa, de Barcelona a Buenos Aires buscando a su hijo

Esta mujer navarra perteneciente a una numerosa familia no ingresó en Leganés hasta los 59 años y fue descrita como una niña "muy independiente, escasamente escolarizada y con dificultades de relación con su madre" (Historia clínica n.XX-1418 recogida en Villasante et al., 2018, p.174-176). Según refiere la historia, decidió salir del convento, donde había ingresado a los 15 años y probablemente había aprendido a escribir, para buscar novio. Su anhelo de independencia le llevó a Barcelona, ciudad en la que trabajó como "sirvienta en casas públicas" (p.174), concibiendo dos hijos; el primero de ellos, internado en la inclusa, falleció de niño.

Según el relato médico, al llevar una "vida muy desordenada" y su segundo hijo "poco atendido", su familia decidió hacerse cargo de él y llevarle a América. Josefa cruzó el Atlántico en 1930, cuando el hijo tenía 10 años, "porque no podía estar sin su hijo", pero la familia regresó a España dos años después, permaneciendo ella en Buenos Aires 12 años más (Villasante et al., 2018, p.174).

En 1944, al año siguiente de regresar de Argentina, Josefa ingresó a petición de su hijo en el manicomio con un cuadro clínico descrito como "psicosis episódica por probable anemia". En su expediente clínico hemos encontrado una carta escrita en varios días de enero (18, 20 y 21) de 1946. En esta carta, dirigida a dos hermanos, relataba su malestar por un episodio de gripe que le generaba una "tos muy profunda", impidiéndole dormir y le obligaba a pasar las noches sentada. Además, describía un ataque epiléptico con estas palabras: "Como a las cuatro perdí el conocimiento, los minutos que estuve gritaron que yo no hoy [*sic*] nada que parecía muerta de amarilla". En la carta que, en general, presenta una desorganización en el relato, Josefa encadenaba diversos temas de preocupación, como la comida, la ropa, su hijo y conversaciones con el médico (Villasante et al., 2018, p.175-176).

A pesar de que la ración diaria estaba consignada en el Reglamento de 1873 (Villasante, 2003, 2008), las quejas por la comida, tanto por escasa como por mal condimentada, se han encontrado con frecuencia en las cartas de Leganés (Villasante et al., 2016, p.122-123). A pesar de ser "pensionista", Josefa pedía patatas, huevos, churrasco, longaniza y "pan blanco"; este era un escaso alimento en la posguerra al que María también aludía. Además, solicitaba un almohadón para "sentarme en el banco duro mi culete flaco" [*sic*], ropa y cosméticos (lápiz labial), argumentando que su hijo "viene cada cuatro meses y no trae ni una botella de agua de Lozoya que también me prueba ya sabe [*sic*] que no se crió conmigo". El último fragmento del texto se lo dedicaba en gran parte a su hijo, a quien reconocía no haber criado y de cuya actitud se lamentaba: "Cuando viene tuerce el pescuezo como un beato y no me cuenta nada". Josefa se mostraba preocupada por él y ansiaba tejerle un chaleco para protegerle "los bronquios", ya que le percibía "muy flaco". Solicitaba "que me traiga las camisas para zurcir" (Villasante et al., 2018, p.176), y es que la dedicación a las labores domésticas y al cuidado de la familia iba a legitimar a la "buena mujer", esa figura de "ángel del hogar", tan promocionada desde el franquismo.

No obstante, poco sabemos de los últimos años de Josefa en Leganés, ya que si bien en el primer mes de ingreso hay dos amplias anotaciones del médico (días 18 y 30), después encontramos apenas otras dos. En 1952, se describía que permanecía tranquila y adaptada al "régimen manicomial", y, en 1962, escribió a su hijo, cuyas visitas fueron muy esporádicas, hasta que falleció, en la institución, en 1965.

## Adela y su copla de despecho

Adela, madrileña y madre de cinco hijos, ingresó en el Manicomio de Leganés a instancias de su marido en 1944 (Historia clínica n.XX-1433 recogida en Villasante et al., 2018, p.177-182). Unos años antes, en 1934, y embarazada de su segundo hijo, presentó dolores ováricos que su marido, farmacéutico, trató con morfina,[10] pero, al interrumpir el tratamiento, Adela lo sustraía de la farmacia, apoderándose de cajas del opiáceo. Ingresó en el Manicomio de Leganés diagnosticada de toxicomanía y personalidad psicopática, cuando el consumo de morfina le llevó a derrochar "gran cantidad de dinero en compras descabelladas y mintiendo descaradamente" (p.177), en una época de posguerra, donde el régimen imponía una política autárquica de austeridad a las familias (Blasco Herránz, 1997, p.87).

El médico, al ingreso, afirmaba que se trataba de una mujer "muy infantil … muy ligera en todas sus cosas, alegre, caprichosa, comunicativa, poco seria en sus apreciaciones y crítica con las cosas" (Villasante et al., 2018, p.177). No obstante, y a pesar de su condición de morfinómana, según recogía el médico en su historia clínica, Adela mostró su desacuerdo con el ingreso que atribuía más a las supuestas relaciones con el amante de su madre que a su adicción. Así, en una carta que dirigía a su marido, le manifestaba su sentimiento de culpa y demanda de perdón:

> Te suplico por tus hijos que me creas. No voy a pedirte que vengas por mí de ninguna manera, porque mi vergüenza por lo ocurrido es cierta, quiero cumplir el castigo que tú me impongas … Sé que he sido ligera y quizás algo peor por lo que todo me condena, pero recuerda las horas felices de nuestro común amor, piensa en nuestros hijos y perdóname (Villasante et al., 2018, p.179).

La súplica de Adela en la que trata de identificarse con la mujer sumisa, buena esposa, dedicada con exclusividad al cuidado de su familia y al ámbito de lo privado (Osborne, 2018), no era sino un modo de legitimar su alta. Un intento de negociación que, independientemente del estado mental de la paciente, fue una verdadera expresión de resistencia (Huertas, 2020b, p.73). Adela vivía las condiciones de la institución como deplorables, y así se las describía a un amigo de la familia, comparándolas con las de los animales, con padecimientos como una colitis, una afección que de manera más generalizada soportaron muchos de los internos. De hecho, la colitis se convirtió en uno de los motivos principales de defunción en el manicomio durante los primeros años de posguerra (Conseglieri, 2013a, p.128).

> No puede hacerse idea lo que es esto, es un sitio de pesadilla. ... Sólo le diré que entre el disgusto el tener los pechos llenos de leche y no poderla sacarla, añádele una colitis enorme, con unos dolores horribles, el pecho enfermo, mi estado moral, y por encima de la falta de todo cuanto tenía en casa un trato que ni a las fieras, pues aún teniendo fiebre aquí como no la bajen, no se puede estar en cama, lo único tumbarse en un patio que es un propio barrizo de cerdos. ¿Se imagina el cuadro?, No de ninguna manera, una cosa es contarlo y otra pasarlo… (Villasante et al., 2018, p.181).

Un documento hallado en la historia que nos resulta especialmente interesante es la letra de una canción titulada "Música de tatuaje" que Adela adaptó de manera autobiográfica a la copla "Tatuaje", compuesta por Xandro Valerio, Rafael de León y Manuel Quiroga en 1941 e interpretada por Conchita Piquer. Esta copla, como otras en plena posguerra, sirvieron como vehículo del dolor y resistencia, contribuyendo a elaborar el duelo de fusilados, exiliados y desaparecidos (Sieburth, 2016). La copla de Adela describe su internamiento en una letra desesperada que bien podrían ser ejemplo de una expresión artística de resistencia al manicomio.

> Me metió en un taxi,
> entre dos loqueros,
> y dijo ¡arreando hacia Leganés!.
> Más por si las moscas,
> mi adorado esposo,
> a venir conmigo no se atrevió él.
> Y vine rabiando, gimiendo y llorosa,
> hasta que por fin llegamos al portal,
> y al verme el portero y otros empleados,
> dijeron a coro ¡una loca más!
> y voy por el pasillo
> Siempre del costurero al comedor...
> (Villasante et al., 2018, p.182).

No podemos confirmar si estas cartas precipitaron la concesión de una licencia de salida a Adela, pero, una vez finalizado dicho permiso, no regresó al manicomio, cursándose el alta definitiva en mayo de 1945.

## Matilde, de la inclusa a madre de familia

Matilde procedente de una inclusa, se quejaba a menudo de sus padres adoptivos, y después de casarse empezó a desconfiar de su marido, separándose en 1915 (Historia clínica n.XX-1461 recogida en Villasante et al., 2018, p.186-192). Según el relato de uno de sus propios hijos en 1945, año del ingreso en el Manicomio de Leganés, le acusaba injustamente al marido de "tener queridas y haber querido violar a sus propias hijas", incluso, de asesinar a una de ellas fallecida de tuberculosis (Historia clínica n.XX-1461, abr. 1945, s.p.). Parece que la suspicacia fue creciendo hasta pensar que le perseguían para asesinarla y arrojarla al agua, por lo que abandonó el domicilio y fue a Madrid, donde vivió sola dedicada a “asistir en casas, cuidar niños”, hasta después de la guerra civil. Una de sus hijas se trasladó a la capital con el propósito de ayudarla, pero poco después también desconfió de ella, convencida que el padre la enviaba para perjudicarle. La situación empeoró, y así se relataba en su historial médico: “Había estado últimamente teniéndome tres meses con agua sola, al principio con un poco de azúcar y después con sal y por fin a última hora sola y calentándola con barreduras de las calles” (Historia clínica n.XX-1461, abr. 1945, s.p.).

Cuando entró en el Manicomio de Leganés como pensionista de primera clase, tenía 71 años y fue diagnosticada de “desarrollo paranoico con delirio de persecución” (Villasante et al., 2018, p.186). En su historial se conservan tres cartas; la primera es una copia mecanografiada dirigida a sus “inolvidables hijos”, escrita en 1946, cuando llevaba un año en la institución:

> Y ahora me tienen entre locos, desnuda, hasta sin camisa, hasta sin comer pues ya llevo un año el veintisiete de abril del pasado mes sin comer más que la triste sopa del mediodía; el café después que vine no lo he visto; leche fresca no la puedo tomar tengo miedo desde un día que me dio T. y me hizo daño y estuve cinco o seis días mala pues no puedo tomar más que condensada; les pido ya que me tienen aquí que me manden condensada (Villasante et al., 2018, p.188).

Otra carta está dirigida en un tono formalista al médico de la institución:

> Señor Don Manuel, muy Señor Mío y de mi mayor consideración. En nombre de Dios le pido con lo más íntimo de mi corazón que por lo que más quiera en este mundo y en el otro, les obligue lo mismo a mi esposo que a mi hija a que me lleven a mi santa casa de donde me sacaron y que me dejen vivir tranquila pues son muchos los años que me tienen así sufriendo, haciendo ver con sus mentiras de que era una mujer de lo más tirado del mundo (Villasante et al., 2018, p.188-189).

En general las cartas a los médicos o directores de la institución, tal como ya hemos referido, intentaban negociar el alta, confiando en la sagacidad del médico para dilucidar que no precisaban ingreso y buscando la complicidad con ellos (Huertas, Villasante, 2019, p.20). En el caso de Matilde se quejaba al médico de cuestiones de la vida cotidiana como la falta de comida o de vestido – “estoy sin ropa no tengo más que una triste bata” (Villasante et al., 2018, p.189) – o, incluso, de maltrato (insultos, amenazas...), sin especificar quienes la maltrataban. Precisamente, Peraita había contribuido a diagnosticar el síndrome parestésico-causálgico, un déficit neuropático debido a las carencias alimentarias de posguerra (Huertas, Del Cura, 2010). Es posible que las pacientes pensaran que el médico no conocía esa escasez o ese trato vejatorio, ya que el control de las cuestiones domésticas descansaba más en las Hijas de la Caridad o en cuidadores, mozos o loqueros, escasamente profesionalizados (Duro Sánchez, Villasante, 2016): “Pues llevo un año sufriendo lo indecible de insultos y amenazas, empujones, hambre pues llevo un año apenas sin comer, pues las comidas no me gustan y por más que hago yo por comer no puedo, pues si algo como, lo devuelvo antes de que me llegue al estómago” (Villasante et al., 2018, p.189). Probablemente esperaba la intercesión de la autoridad médica para mejorar las condiciones de habitabilidad y, sin duda, en última instancia, su alta. No sabemos si los reproches a sus hijos por el traslado al manicomio o las plegarias al médico surtieron efecto, pero a Matilde se le concedió, a petición de su hijo, una salida temporal de tres meses, período máximo de la licencia temporal contemplado en el Reglamento de 1941 (Conseglieri, Villasante, 2007, p.128).

## Elvira, de maestra a poeta

Elvira, nacida en un pueblo castellano, había sido maestra, una profesión que a diferencia de otras como la diplomacia, la administración de la justicia o la policía (Roca i Girona, 2005), era aceptada para las mujeres en el franquismo. Soltera trabajó en diferentes pueblos de Castilla y León, hasta que, en 1932, en la escuela donde ejercía comenzaron a notar que su rendimiento docente empezaba a decaer y fue apartada de su puesto. Ya "jubilada" decidió trasladarse a Madrid, cambiando de domicilio en varias ocasiones, según consta en su historial médico, por problemas de convivencia con los vecinos (Historia clínica n.XX-1574 recogida en Villasante et al., 2018, p.198-202).

Uno de sus hermanos la llevó a Leganés y la recordaba como una niña nerviosa que presentaba ataques "de tipo histérico" por disgustos de cualquier tipo. Poco antes de llegar al manicomio, el hermano encontró a la paciente "abandonada, comiendo mal y consumiendo abundante alcohol". Cuando, en 1947, entró en Leganés, tenía 61 años y estaba diagnosticada de un "síndrome paranoide", que no le impidió mostrar cierta vocación poética. Dos años después de su ingreso, dedicó un poema a su hermano "capitán de transmisiones" y una segunda composición poética, "Romeo y Julieta", que reflejaba aptitudes y conocimientos literarios propios de una mujer instruida y que firmaba, en 1949, como "Poetisa Castellana. Ex maestra nacional" (Villasante et al., 2018, p.200).

La única carta conservada, firmada en 1950, estaba dirigida al juez (instancia reconocida en el decreto de 1931 como legitimadora del ingreso), a través del director médico del manicomio, y en ella solicitaba "que me saque inmediatamente de aquí", una expresión repetida en los escritos de otras internas, y que terminaba con desesperación y rabia: "Así que espero mi salida inmediata sin restricciones ni tonterías mal hechas con descaro" (Villasante et al., 2018, p.199).

> Para el Señor Juez de Getafe. Señor Director Médico del manicomio de Leganés. Señor, ayer sostuve una conversación con el Administrador de este establecimiento en la oficina de aquí, y deduje que si en la vida fuera de aquí, en mi vida profesional, tuve enemigos ocultos y a veces descarados, que privaron a las niñas de España de una gran Maestra. … Los motivos que atañen para retenerme son superfluos, no llegan a la categoría de motivos serios para encerrar a una señora en un manicomio, ni menos para retenerla tres años en él. ... Aquí se está dando lugar a que pierda la salud excelente que siempre tuve, y se me olviden los conocimientos que adquirí con tanto trabajo, que muchas personas quisieran haber tenido (p.201-202).

Esta misiva, redactada tres años después de su internamiento, no tuvo el éxito que su autora esperaba, ya que permaneció 15 años en la institución, siendo trasladada a un asilo de Ávila posteriormente.

## Mujeres resistiéndose a vivir en el Manicomio de Leganés

Asumiendo que solo hemos trazado fragmentos de las vidas de María, Elena, Josefa, Adela, Matilde y Elvira, trataremos de recopilar algunos elementos tanto comunes como dispares entre ellas para enriquecer la historia del Manicomio de Leganés con esta visión "desde las pacientes". Todas ellas, a pesar de sus diferencias de edad (de los 37 de Elena a los 71 de Matilde) y de procedencias diversas de la geografía española (Cáceres, Madrid, Castilla y León…), ingresaron en un Manicomio Nacional sito a 14 kilómetros de Madrid, que había sido fundado en 1852 como una pequeña Casa de Dementes para alojar a pacientes pobres a cargo de la Beneficencia General del Estado (Peset, 1995; Villasante, 2008). Sin duda, la dificultad para llegar a esta localidad madrileña en una época de dura posguerra se traduce en las cartas que expresan la añoranza de las visitas de las propias familias. Este pequeño establecimiento había crecido progresivamente hasta que en la primera década después de la guerra civil, momento en el que nuestras protagonistas ingresaron allí, había en torno a cuatrocientas personas (Conseglieri, 2013a).

Un rasgo común fue su alojamiento como pensionistas o "distinguidas" (Villasante, Candela, 2022), un grupo selecto, más pequeño durante el siglo XIX, que se incrementó durante la autarquía franquista favoreciendo la entrada de enfermos "de pago" (hasta 1952 ingresaron casi el doble de pensionistas), para mitigar la escasa asignación económica aportada por la administración sanitaria. En cualquier caso, dado el elevado índice de analfabetismo en España, mayor aún en las mujeres, la escritura era minoritaria entre ellas y solo al alcance de algunas privilegiadas. El mayor índice de instrucción académica estaba asociado a una mejor situación económica, cuestión que introduce un sesgo en este grupo cuyo nivel cultural era superior (especialmente marcado en Elena) al de muchas de sus compañeras "pobres".

En aquella década, las mujeres ingresaron en mayor porcentaje que los hombres (52%) (Conseglieri, 2013a), siendo frecuentemente solteras y dedicadas a "sus labores", salvo el caso de Elvira, que constituye una excepción. Muchas de ellas se quedaban hasta su fallecimiento dentro de la institución, como Elena, o bien, eran trasladadas a otros establecimientos, pero aún en el caso de salir relativamente pronto (algo menos de un año), como Adela, el manicomio era percibido como un espacio de exclusión. Las preguntas de Adela "¿tú sabes dónde me has enviado?, ¿tú tienes idea siquiera lo que es un manicomio?" (Villasante et al., 2018, p.180), o la expresión de Matilde "y ahora me tienen entre locos" (p.188), denotan esa ubicación paradójica o esa suerte de paratopía que supone estar en la institución separadas de sus hogares (Huertas, 2020b, p.68).

El ingreso requería la autorización del director general de la beneficencia y obras sociales y debía adjuntarse un certificado firmado por un médico que generalmente era solicitado por los familiares, quienes mediatizaban en el internamiento tal como hizo el hijo de Josefa o el hermano de María o de Elvira, una circunstancia común también señalada en otras instituciones (Prestwich, 1994). En el caso de Adela, fue su marido a quien rogaba su salida, con cierto reproche, en una súplica que mezclaba el arrepentimiento y el anhelo de perdón ("quiero cumplir el castigo que tú me impongas") (Villasante et al., 2018, p.179). Un marido que, responsable del tratamiento con morfina, se había acogido al decreto de julio de 1931 para solicitar su ingreso: "La enfermedad psíquica que aconseje su aislamiento, la peligrosidad de origen psíquico, la incompatibilidad con la vida social, las toxicomanías incorregibles que pongan en peligro la salud del enfermo o la vida o los bienes de los demás" (Decreto…, 7 jul. 1931, p.188). De hecho, la "peligrosidad de origen psíquico" fue el supuesto legal más utilizado para ingresar a estas seis mujeres; conceptos ambos, el de peligrosidad y enfermedad mental, que han estado sujetos a debate por juristas y psiquiatras (Campos, 2021). Esta peligrosidad se remarca aún más en Elena o Adela (trato desenfadado con varones, gastos excesivos, mentiras), y sus ingresos trataban de corregir aquellas conductas consideradas impropias para ellas. Esta idea ha sido señalada por Augusta Molinari (2005, p.383) en relación a las mujeres institucionalizadas en el Hospital Psiquiátrico Quarto di Mare (Génova) en los siguientes términos: "El internamiento tenía como objetivo primario reconducir el comportamiento femenino a un ámbito socialmente aceptado". Los comportamientos que trasgredían la normatividad doméstica requerían imponer corrección y disciplina a la trasgresora, tal como ha destacado Ríos Molina (2009, p.153) en el estudio de La Castañeda, México.

Las demandas de salida a los familiares o a los médicos les llevaban a ritos de subordinación como el verbalizado por Elena ante el médico – trabajar sin cobrar – (Huertas, Villasante, 2019, p.21): "Si me quiere buscar una colocación yo trabajaré para usted sin cobrar ni un céntimo". Una relación médico/paciente que se traducía en una "negociación tensa" o dialógica (Rivera Garza, 2022, p.43), cuestionada sin embargo por otras investigadoras (Gorbach, 2011; Yébenes, 2016). La subordinación hacia el marido es fácil de observar en Adela – "empezaré diciéndote que ayer día de la virgen confesé y comulgué y tú ya sabes lo que eso significa en mí que jamás lo hice" (Villasante et al., 2018, p.178) –, convirtiéndose en actitudes o instrumentos de negociación para abandonar el manicomio. La asunción o buena disposición hacia las labores maternales y domésticas, responsabilidades ineludibles de las mujeres (Morcillo Gómez, 2015), también se establece como moneda de cambio. "Anúlame de tu vida" era el comienzo de una carta escrita al marido en 1944 que continuaba: "Pero ¡por Dios! Déjame al lado de mis hijos. Déjame cuidar nuestra casa y atenderlos a ellos que siempre lo hice con esmero" (Villasante et al., 2018, p.180).

Más allá de la función de control social de las instituciones, sobre la que hay extensa literatura pero que escapa al objetivo de nuestro trabajo, nos interesa el control de la correspondencia de la cual las mujeres eran conscientes (Villasante, 2018). Esta práctica, generalizada en otras instituciones (Barfoot, Beveridge, 1990; Montanari, 2015, p.139), también fue ejercida en Leganés desde su fundación (Villasante, Candela, 2022) y se reflejó en el Reglamento (1941) prohibiendo explícitamente a los empleados entregar cartas. Adela era bien conocedora de ello: "Ésta la escribo con la autorización de la superiora quien sabe que eres un buen amigo nuestro" (Villasante et al., 2018, p.181). De hecho, las Hijas de la Caridad no se ocupaban solo del cuidado, sino también de la administración y vigilancia de las internas, viéndose su autoridad y control reforzado con la llegada del franquismo, favorecido por una enfermería escasamente profesionalizada. No es extraño que la censura tratara de evitar que las críticas sobre las condiciones de alojamiento llegaran a las familias que pagaban la estancia, porque, sin duda, una de las quejas más frecuentes y repetida en las cartas, no solo de las mujeres, era el trato, las comidas o el mal estado de la ropa; muy evidentes en cartas como las de Matilde que escribe: "Desnuda, hasta sin camisa, hasta sin comer … más que la triste sopa del mediodía". O la de Adela que dice: "Aquí no hay nadie ni siquiera medio razonable. Luego sin agua, la comida renuncio a explicársela" (citadas en Villasante et al., 2018, p.180). Todos estos lamentos, en el fondo, traducían un deseo último: no querer pertenecer ni estar en el manicomio.

## Consideraciones finales

Conocer las vidas de estas seis mujeres del Manicomio Nacional de Santa Isabel de Leganés (España) nos permite acercarnos solo someramente a sus vidas y experiencias de internamiento. Como estudios de caso conocemos su limitación, pero las hemos rescatado porque suponen ejemplos de voces apagadas durante décadas que pueden ilustrar la doble condición subalterna que supone ser "mujer" y estar "loca". El dolor, el hambre, la pena, el sufrimiento, la rabia y la impotencia son algunos de los sentimientos que captamos en estas letras marginadas en el espacio manicomial, hasta ahora silenciadas.

Las cartas no enviadas, sobre las que se ejercía un control, nos muestran los discursos no aceptados y censurados que debían moldearse y modificarse para conseguir, a través de suplicas, mandatos o intentos de negociación, salir del manicomio y obtener la libertad. Consideramos que las letras de María, Josefa, Elena, Adela, Matilde o Elvira ayudan a construir la historia de la locura "desde abajo", pero sin duda aún hay una gran laguna historiográfica que posteriores investigaciones sobre el Manicomio Nacional de Leganés (España) u otros establecimientos psiquiátricos deberían contribuir a rellenar.

## NOTAS

[1] El "Programa Leganés" ha consistido en una estrategia de investigación conjunta y metodología común para cuatro tesis doctorales que han utilizado las historias clínicas (primera mitad del siglo XX) del Archivo Histórico del Hospital Universitario José Germain, Leganés, Madrid (en adelante HUJG; antes de 2020 la institución se denominaba Instituto Psiquiátrico José Germain), para analizar la historia del establecimiento, su funcionamiento institucional, las características de la población manicomial y las prácticas psiquiátricas. Estas tesis analizan el Manicomio Nacional de Leganés en diferentes periodos políticos: el primer tercio del siglo XX (Candela, 2017); la Segunda República (Tierno, 2019); la guerra civil (Vázquez de la Torre, 2012) y la autarquía franquista (Conseglieri, 2013a). Véase: http://psiquiatriaycambiosocial.com/es/programa-leganes/.

[2] Si bien las cartas originales se hallan en la historia clínica (historias clínicas n.1220, 1303, 1418, 1433, 1461 y 1574, en el Archivo Histórico del HUJG, Leganés), los fragmentos – entrecomillados o no – de las de cartas, citados en el texto, se pueden consultar en la recopilación *Cartas desde el manicomio* (Villasante et al., 2018).

[3] Era habitual que la familia fuese quien aportaba la información sobre quiénes y cómo eran las pacientes y por tanto quien reconstruía la historia de vida de éstas (Conseglieri, Baquero, 2021).

[4] En Leganés se ha documentado su utilización en los años 1930; véase Candela (2017, p.463-464) y Tierno (2019, p.255).

[5] En su historia se recoge que en el Sanatorio Psiquiátrico-Neuropático había sufrido "siete accesos febriles de paludismo terapéutico y varios tratamientos generales con arsenicales y Pernemon". De hecho, Gonzalo Rodríguez Lafora fue el primer neuropsiquiatra que utilizó la malarioterapia en España. En el sanatorio que él dirigía se realizaron más de trecientas paludizaciones, una técnica popularizada por el Nobel Julius Wagner von Jauregg (1922).

[6] José Miguel Sacristán (1887-1957) fue director de este manicomio regentado por las Hermanas Hospitalarias, más conocido como Sanatorio de Mujeres de Ciempozuelos, desde 1919 hasta el comienzo de la guerra civil.

[7] José Salas Martínez (1905-1962), padre de la prestigiosa científica Margarita Salas, publicó en 1935 el primer manual dedicado a enfermeras psiquiátricas, *Manual de la enfermera general y psiquiátrica*.

[8] El coma insulínico, la cardiazolterapia y el electrochoque formaban parte de las terapias de choque que alcanzaron gran difusión en las instituciones psiquiátricas en los años 1940 (Shorter, 1997).

[9] Se ha barajado la posibilidad que el "amigo Valentín" fuese Valentín Conde López, un médico que trabajó en los años 1960, siendo director entre 1970 y 1973 (Desviat, 2002).

[10] Algunas autoras han señalado como la psiquiatría de la primera mitad del siglo XX se nutrió de otras especialidades, como la ginecología, para gestar un discurso psiquiátrico hegemónico (García Díaz, Jiménez-Lucena, 2023). En el caso de Adela, incluso su ginecólogo le llegó a diagnosticar de "infantilismo de matriz" (Villasante et al., 2018, p.177).
